# Risk Factors and Management of Catheter Malfunction During Urgent-Start Peritoneal Dialysis

**DOI:** 10.3389/fmed.2021.741312

**Published:** 2021-11-01

**Authors:** Lijuan Zhao, Jun Yang, Ming Bai, Fanfan Dong, Shiren Sun, Guoshuang Xu

**Affiliations:** Department of Nephrology, Xijing Hospital, The Fourth Military Medical University of People's Liberation Army, Xi'an, China

**Keywords:** urgent-start peritoneal dialysis, catheter malfunction, surgeon, colonic dialysis, serum potassium

## Abstract

**Background:** Catheter malfunction is a common complication of peritoneal dialysis (PD). This study aimed to retrospectively analyze the risk factors and management of catheter malfunction in urgent-start PD.

**Methods:** Patients who underwent urgent-start PD were divided into catheter-malfunction and control groups. Baseline demographic and laboratory data of the two groups were compared, and the risk factors for catheter malfunction were analyzed. Primary outcome measure was catheter survival, and the secondary outcomes were surgical complications and malfunction treatment.

**Results:** Total of 700 patients was analyzed, among whom 143 (20.4%) experienced catheter malfunctions, specifically catheter migration (96, 67.1%), omental wrapping (36, 25.2%), and migration plus omental wrapping (11, 7.7%). Catheter survival time in the malfunction group (202.5 ± 479.4 days) was significantly shorter than that in the control group (1295.3 ± 637.0 days) (*P* < 0.001). Multivariate analysis revealed higher body mass index [hazard ratio (HR), 1.061; 95% confidence intervals (CI), 1.010–1.115; *P* = 0.018], lower surgeon count (HR, 1.083; 95% CI, 1.032–1.136; *P* = 0.001), and higher serum potassium (HR, 1.231; 95% CI, 1.041–1.494; *P* = 0.036) as independent risk factors for catheter malfunction, while older age (HR, 0.976, 95% CI, 0.962–0.991; *P* = 0.002) and colonic dialysis (HR, 0.384; 95% CI, 0.254–0.581; *P* < 0.001) as protective factors. Further subgroup analysis revealed a shorter catheter survival time in patients with younger age ( ≤ 40 years), higher serum potassium levels (≥5 mmol/L), while a longer catheter survival time in patients with colonic dialysis. PD tube and subcutaneous tunnel preservation was successful in 41 out of 44 patients with omental wrapping. All patients had good post-incision prognoses.

**Conclusions:** Urgent-start PD is safe and effective for unplanned PD patients. Adequate pre-operative colonic dialysis and serum potassium level control are conducive in preventing catheter malfunction. Conservative treatment is effective in managing catheter migration alone, while preservation of the PD tube and the subcutaneous tunnel is effective for omental wrapping.

## Introduction

End-stage renal disease (ESRD) [i.e., stage 5 chronic kidney disease (CKD)] has become a major public health burden worldwide. The incidence of ESRD continues to increase owing to the greater prevalence of CKD and diabetes mellitus patients (1) and high perioperative morbidity and mortality are significantly higher in ESRD patients due to multiple comorbidities (2). Peritoneal dialysis (PD) is an important renal replacement therapy (RRT) for ESRD patients (3). Catheter insertion was recommended at least 2 weeks before initiating PD (4, 5). However, for these patients, delaying PD for 2 weeks is unrealistic. Urgent-start PD is warranted in newly diagnosed ESRD patients who have not been on dialysis and need RRT initiation within 2 weeks (6). Urgent-start PD has been suggested as a feasible and well-tolerated alternative to hemodialysis, reducing the risk of central venous catheter-related complications, such as central venous stenosis, bacteremia, and thrombosis related to temporary hemodialysis use (7, 8).

The success of PD mainly depends on a well-functioning peritoneal catheter (9). Catheter-related complications frequently cause PD failure, requiring session delays, or even permanent procedure changes. Catheter-related complications are responsible for up to 20% of all permanent transfers to HD (10, 11). Catheter malfunction, characterized as mechanical failure in dialysate inflow or outflow, is a common complication of PD, forcing conversion to hemodialysis (12, 13). Approximately 4–20% of PD patients may have catheter malfunction, affecting the overall survival rate, and quality of life (14, 15). The risk of catheter malfunction might be limiting the widespread use of urgent-start PD (16). Although urgent-start PD-associated complications have been reported, the evidence is relatively weak due to regional differences and limited sample sizes (17), and urgently starting PD after catheterization has not been associated with further catheter dysfunction or other complications (18, 19). To improve the clinical application of urgent-start PD, it is important to investigate the risk factors and management of catheter malfunction.

Our center has had more than 700 PD patients, and all patients experienced urgent catheter insertion and immediate PD initiation. In this study, we retrospectively analyzed the characteristics of patients receiving urgent-start PD, as well as the risk factors, management methods, and prognosis for catheter malfunction.

## Materials and Methods

### Study Population

This retrospective cohort study enrolled patients who underwent urgent-start PD at our institution between January 2013 and December 2019. The inclusion criteria were as follows: patients aged ≥16 years who required Tenckhoff catheter insertion for long-term PD. The exclusion criteria were as follows: age <16 years, extubation withdrawal for other reasons (e.g., thoracoabdominal fistula and hernia), loss to follow-up, hemodialysis self-withdrawal, or death within 1 month of catheterization. All patients were followed up for at least 6 months. This study was approved by the Ethics Committee of Xijing Hospital and informed consent was waived because of the retrospective study design.

### PD Program

**Figure 1:**
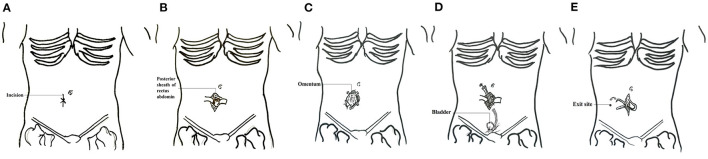
Schematic diagram of surgical technique of PD placement. **(A)** Location of the incision. **(B)** The skin was incised and separated layer by layer to the posterior sheath of the rectus abdominis. **(C)** The posterior sheath was then cut and a purse suture was made. **(D)** Insertion of the catheter and ligating the pox. The internal cuff was secured within the rectus muscle. **(E)** A subcutaneous tunnel was established and the incision was closed.

Before catheter insertion, urinary bladder emptying, and skin preparation using a povidone-iodine solution and standard draping were performed. Prophylactic intravenous cefazolin was routinely administered, except in patients with penicillin or cephalosporin allergies. An open approach through a small paramedian paraumbilical incision was performed in all patients. Only straight double-cuff Tenckhoff dialysis catheters were used. An arc-shaped subcutaneous tunnel was established from the outer top to the outer bottom of the incision, and the catheter was pulled out from the outer lower outlet of the incision (Figure 1). PD was then initiated as early as 24 h after catheter placement. The dosage was gradually transferred from 300 to 2,000 mL each time until 3 × 2,000 mL on the 7th day. Flushes were started using heparin saline within 1 week of placement to help prevent early catheter obstruction from fibrin plugs or clots.

### Colonic Dialysis

Each colonic dialysis session usually lasted 1 h, and the total volume of dialysate was 8–10 L. The colonic dialysate was performed with concentrated dialysate A (catalog no. WGTXF-2, prepared by Weigao company), dialysate B (catalog no. WGTXF-2F, prepared by Weigao company), and ultrapure water (prepared by Weigao company) in a ratio of 1:1.225:32.775. The dialysate temperature was warmed to 34–38°C and delivered using a v 5.3.1 Colonic Therapy System (Model JS-308F, Jinjian Medical Instrument Co., Ltd., Guangzhou, China). Patients were asked to empty their bladder before dialysis to reduce discomfort. During dialysis, patients were asked to remain in a left recumbent position with their two knees bent. A single-use double-lumen rectal catheter (Kerui Medical Equipment Trading Co., Ltd., Zhengzhou, China) was inserted through the anus into the colon to an intubation depth of 65–75 cm, reaching the ascending colon. The colonic dialysate was irrigated in an impulse type into the colon through the inner cavity for 10 s and suspended for 15 s. After allowing the dialysate to remain in the patient for 8–10 min, the solution and wastes were drained out of the colon through the external cavity for 18–20 s. Both cavities had sided holes to prevent blockage. During the procedure, the dialysate was changed repeatedly until the end of dialysis, and the pressure in the lumen was 50–65 kPA during irrigation and 3–8 kPA during drainage.

### PD Catheter Malfunction and Management

**Figure 2:**
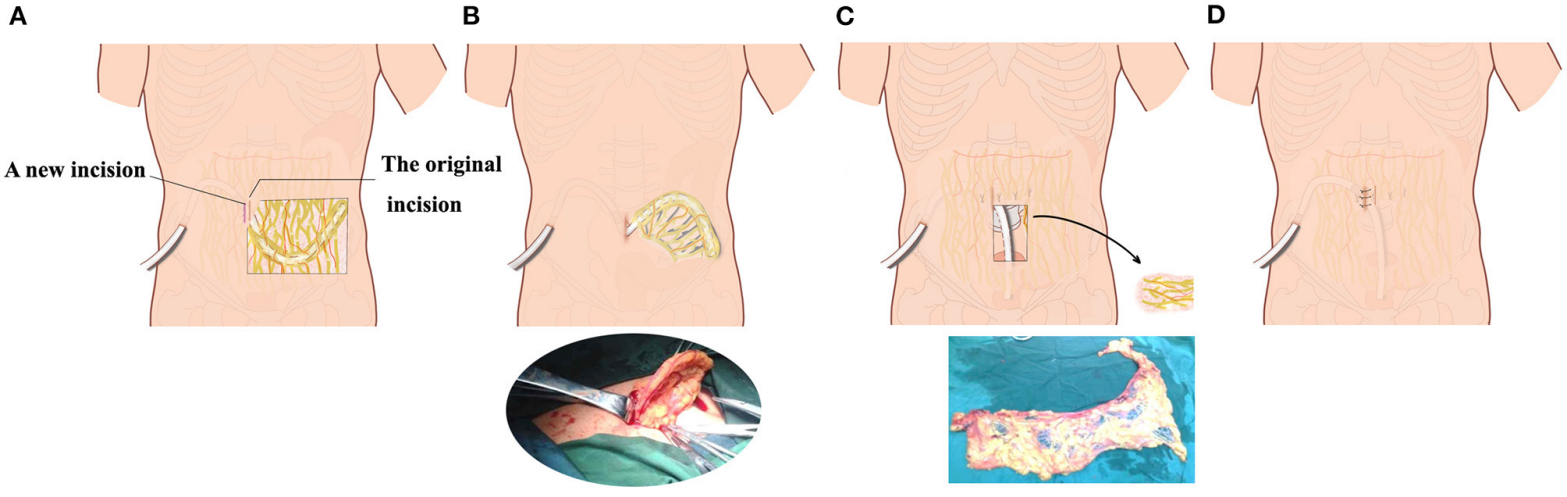
Schematic diagram of the second operation with preservation of peritoneal dialysis catheter and subcutaneous tunnel. **(A)** A second incision was selected lateral to or medial to the original abdominal incision in the omentum wrapping patient. **(B)** The internal peritoneal segment of the peritoneal dialysis catheter and part of the omentum were pulled out from the surgical incision. **(C)** After partial excision of omentum around the catheter, the catheter was re-inserted into the pelvic cavity. **(D)** The peritoneum, anterior sheath of rectus abdominis, subcutaneous tissue, and skin were sutured layer by layer.

Catheter malfunction refers to drainage failure, or the inability to drain peritoneal dialysate effluent reliably within 45 min (20). Catheter tip migration and omental wrapping are the most common types of catheter malfunction (21). Catheter tip migration was characterized by tip location above the pelvic brim on abdominal radiographs and inability to drain the dialysate effluent reliably within 45 min (22). Tissue plasminogen activator (tPA) injection through the catheter was attempted to clear the catheter of clots or fibrin plugs. Catheter malfunctions were managed conservatively, including moderate physical activity (walking on stairs and jumping slightly), intestinal and bowel relaxation, manual reduction, second operation (catheter repositioning or reinsertion, either by an open surgical method), and extubation. Second operations were open surgeries under local anesthesia. A longitudinal incision was made lateral or medial to the original incision. After lidocaine infiltration, layer-by-layer dissection into the rectus abdominis was performed. After the peritoneal incision, the abdominal segment of the peritoneal dialysis tube was removed. If there was omental wrapping, the omentum was separated and ligated, and the redundant omentum was removed. Then, the PD tube was placed again into the pelvis with oval forceps (Figure 2).

### Baseline Demographic and Laboratory Data, and Study Outcomes

Demographic and clinical data included sex, age, height, weight, body mass index, blood pressure, occupation, educational degree, primary kidney disease, and history of abdominal surgery, pre-operative colonic dialysis, or enema. Baseline laboratory data were also evaluated before the PD catheter. Moreover, the indications for PD catheter insertion, date of insertion, surgeon, indication for catheter removal (including obstruction, infection, kidney transplant, functional recovery, or patient mortality), and time from placement to removal or obstruction were also collected.

The primary outcome measure was catheter survival, which was defined as the time that a PD catheter could be preserved after insertion before it had to be abandoned because of infection or mechanical malfunction. Two of our authors collected and entered the data simultaneously. Disagreements were resolved through discussion. The secondary outcomes were surgical complications and malfunction treatment outcome.

### Statistical Analysis

Continuous and categorical variables were expressed as means with standard deviations (SD) and event numbers with percentages, respectively. Normally distributed continuous variables were compared using a *t*-test; otherwise, a Mann-Whitney rank test was employed. Categorical variables were analyzed using the chi-square or Fisher's exact tests. Independent risk factors, such as age, sex, height, weight, blood pressure, occupation, educational degree, primary disease, previous abdominal surgery, surgeon, and serum electrolyte, creatinine, albumin, alkaline phosphatase, and blood urea nitrogen levels, were evaluated using the Cox regression model. Collinearity diagnosis and relation analysis were conducted, and only one of the variables with significant correlation (*P* < 0.05) was included in the multivariate analysis. Patient baseline characteristics and major endpoints were available for all included patients. Mean imputation was employed for the missing data. Statistical analysis was performed using the SPSS 16.0 software package. A two-tailed *P* < 0.05 was considered statistically significant.

## Results

### Baseline Characteristics

**Figure 3:**
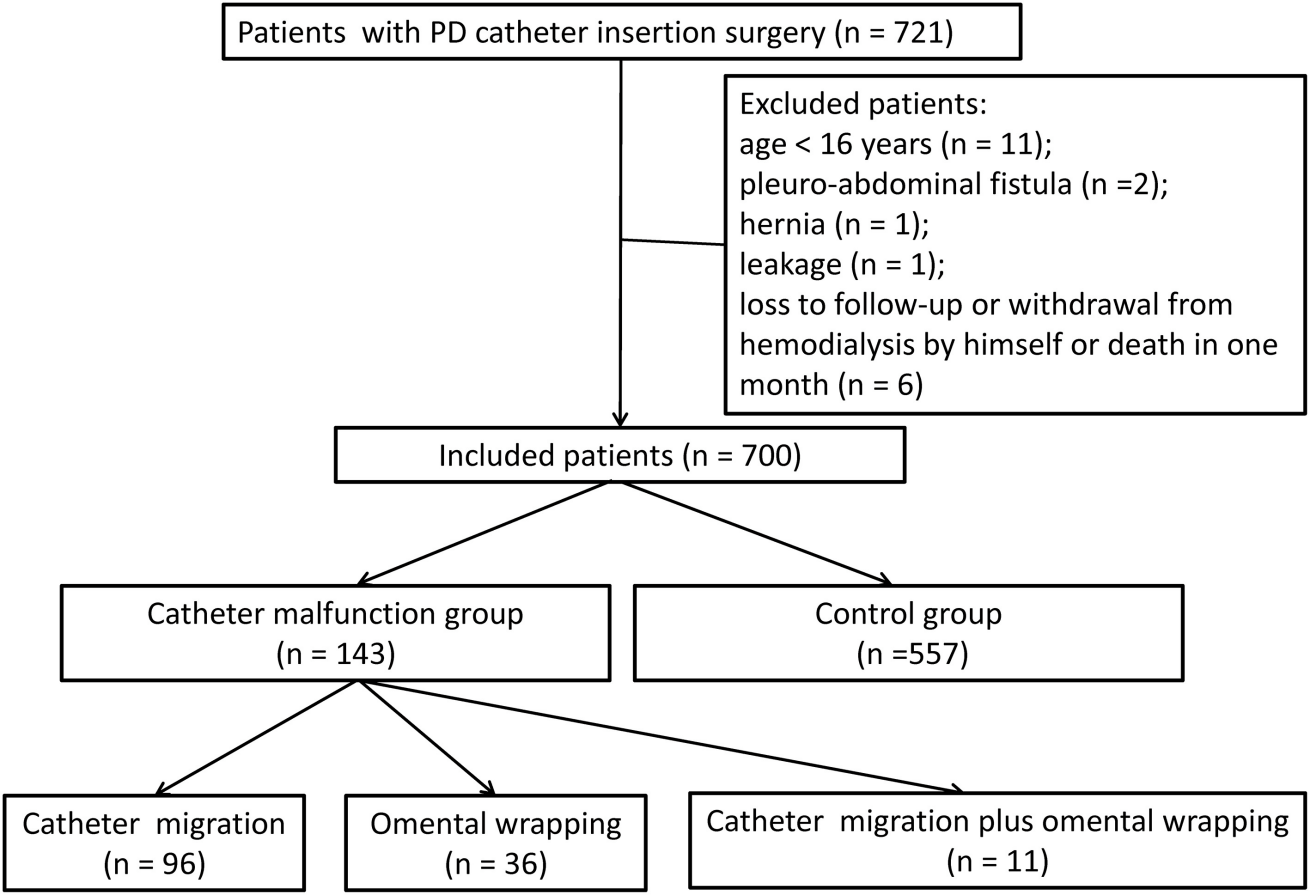
Inclusion flowchart of patients with urgent-start peritoneal dialysis.

From January 2013 to December 2019, 721 patients underwent new PD catheterization and conventional open surgery. Eleven patients aged <16 years, two patients with thoracoabdominal fistula, one patient with indirect inguinal hernia, one patient with scrotal effusion, and six patients who were lost to follow-up/self-converted to HD/withdrew/died within 1 month after catheterization were excluded. A total of 700 patients with new PD were enrolled, including 143 (20.4%) with catheter dysfunction (displacement/omental wrapping) (malfunction group) and 557 (79.6%) without catheter dysfunction (control group) (Figure 3).

The baseline characteristics of the included patients are presented in Table 1. The two groups showed significant differences in age (*p* < 0.001), body mass index (*p* = 0.028), diastolic blood pressure (*p* = 0.002), primary kidney disease (*p* = 0.002), pre-operative colonic dialysis (*p* < 0.001), and surgeon (*p* < 0.001). In addition, there were significant differences between the two groups in serum uric acid (*P* = 0.030) and serum potassium (*P* = 0.011) before catheterization (Table 2).

**Table 1:** Demographic characteristics and clinical data of the study population.

**Variables**	**Malfunction group (*n* = 143)**	**Control group (*n* = 557)**	***P*-value**
Sex (male, %)	87, 60.8%	322, 57.8%	0.568
Age (years)	38.5 ± 14.6	45.0 ± 15.1	<0.001
Height (cm)	165.9 ± 8.0	166.4 ± 7.9	0.495
Weight (kg)	61.7 ± 10.2	60.3 ± 9.9	0.127
Body mass index (kg/m^2^)	22.3 ± 2.7	21.7 ± 2.9	0.028
Systolic pressure (mmHg)	147.1 ± 20.3	144.5 ± 15.3	0.138
Diastolic pressure (mmHg)	92.8 ± 13.8	89.0 ± 12.8	0.002
**Occupation (** ***n*** **, %)**			0.054
Farmer	94, 65.7%	404, 72.5%	
Student	7, 4.9%	10, 1.8%	
Worker and others	42, 29.4%	143, 25.7%	
**Degree of education (*****n**,* **%)**			0.083
Elementary school and below	46, 32.2%	224, 40.2%	
Senior school and above	97, 67.8%	333, 59.8%	
**Primary kidney disease (** ***n*** **, %)**			0.002
Primary glomerular diseases	124, 86.7%	408, 73.2%	
Diabetes	11, 7.7%	97, 17.4%	
ADPKD	1, 0.7%	6, 1.1%	
Obstructive nephropathy	2, 1.4%	1, 0.2%	
Others	5, 3.5%	45, 8.1%	
History of abdominal operation (*n*, %)	15, 10.5%	81, 14.5%	0.223
Pre-operative colonic dialysis (*n*, %)	81, 56.6%	483, 86.7%	<0.001
**Surgeon (** ***n*** **)**			<0.001
0	4	5	
1	3	3	
2	2	26	
3	2	61	
4	48	169	
5	12	108	
6	0	5	
7	11	5	
8	3	17	
9	11	16	
10	9	17	
11	12	111	
12	26	14	

*ADPKD, autosomal dominant polycystic kidney disease*.

**Table 2:** Laboratory data before PD catheter insertion.

**Variables**	**Malfunction group (*n* = 143)**	**Control group (*n* = 557)**	***P*-value**
White blood count (10^9^/L)	6.2, 2.4	6.3, 2.4	0.636
Hemoglobin (g/L)	89.3, 23.0	92.2, 22.3	0.160
Red blood count (10^12^/L)	3.0, 0.7	3.3, 6.4	0.692
Hematocrit value	28.9, 14.8	28.7, 11.6	0.689
Blood platelet (10^12^/L)	156.7, 66.2	167.7, 66.3	0.078
BUN (mmol/L)	29.2, 34.9	26.9, 29.9	0.426
Serum creatinine (umol/L)	777.4, 371.2	719.5, 410.9	0.128
Serum uric acid (umol/L)	379.6, 123.7	409.0, 142.8	0.030
Serum total protein (g/L)	60.1, 9.3	61.6, 8.7	0.081
Serum albumin (g/L)	36.6, 7.1	36.2, 6.5	0.505
Serum potassium (mmol/L)	4.7, 0.8	4.5, 0.8	0.011
Serum sodium (mmol/L)	139.5, 12.2	140.5, 3.9	0.093
Serum chlorine (mmol/L)	103.0, 4.9	102.2, 5.1	0.070
Serum calcium (mmol/L)	2.0, 0.3	2.0, 0.8	0.480
Serum phosphorus (mmol/L)	1.9, 0.5	1.8, 0.6	0.187
Serum iPTH (pg/ml)	319.5, 286.5	297.6, 212.6	0.319
Serum cholesterol (mmol/L)	4.3, 1.3	4.3, 1.1	0.808
LDL (mmol/L)	2.6, 1.0	2.6, 0.9	0.788
Serum triglycerides (mmol/L)	1.7, 1.1	1.7, 1.4	0.747

*PD, peritoneal dialysis; iPTH, intact parathyroid hormone; LDL, low-density lipoprotein*.

### Analysis of Risk Factors for PD Catheter Malfunction

The risk factors for PD catheter malfunction were analyzed using univariate and multivariate Cox regression analyses. In the univariate analysis, age (*p* < 0.001), occupation (*p* = 0.030), body mass index (*p* = 0.028), diastolic pressure (*p* = 0.004), primary kidney disease (*p* = 0.024), pre-operative colonic dialysis (*p* < 0.001), surgeon (*p* = 0.008), and serum potassium level (*p* = 0.008) were significantly associated with catheter dysfunction (Table 3). Multivariate analysis revealed that higher body mass index [hazard ratio (HR), 1.061; 95% confidence interval (CI), 1.010–1.115; *P* = 0.018], lower surgeon count (HR, 1.083; 95% CI, 1.032–1.136; *P* = 0.001), and higher serum potassium level (HR, 1.231; 95% CI, 1.014–1.494; *P* = 0.036) were independent risk factors for catheter malfunction, while older age (HR, 0.976; 95% CI, 0.962–0.991; *P* = 0.002) and colonic dialysis (HR, 0.384; 95% CI, 0.254–0.581; *P* < 0.001) were protective factors (Table 3). There was no significant difference in the incidence of peritoneal dialysis-related peritonitis between patients with aged >40 and ≤ 40 years (132/421 vs. 69/279, *P* = 0.058), nor between the colonic dialysis and no colonic dialysis groups (168/564 vs. 33/136, *P* = 0.245, data not shown).

**Table 3:** Univariate and multivariable Cox regression analyses of the risk factors of PD catheter malfunction.

**Variables**	**Univariate analysis**			**Multivariate analysis**		
	**HR**	**95%CI**	***P*-value**	**HR**	**95%CI**	***P*-value**
Age	0.957	0.945–0.970	<0.001	0.976	0.962–0.991	0.002
Occupation	1.397	1.034–1.888	0.030			
Body mass index (kg/m^2^)	1.059	1.006–1.115	0.028	1.061	1.010–1.115	0.018
Diastolic pressure	1.017	1.005–1.028	0.004			
Pre-operative colonic dialysis	0.241	0.173–0.336	<0.001	0.384	0.254–0.581	<0.001
Primary kidney disease	0.749	0.583–0.963	0.024			
Surgeon	1.067	1.017–1.120	0.008	1.083	1.032–1.136	0.001
Serum potassium	1.286	1.067–1.551	0.008	1.231	1.014–1.494	0.036

*PD, peritoneal dialysis; HR, hazard ratio; CI, confidence interval*.

### Types of PD Catheter Malfunction

**Figure 4:**
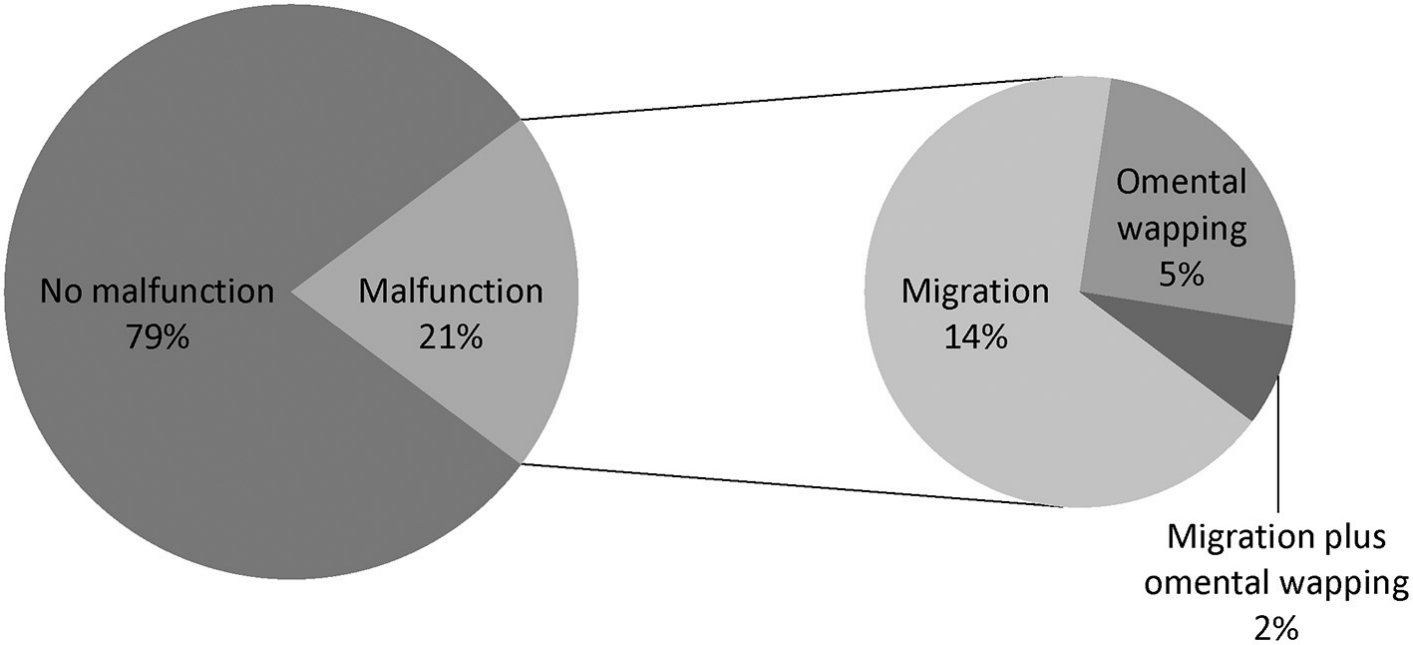
Catheter dysfunction types in urgent-start peritoneal dialysis.

The types of catheter malfunction included catheter migration (*n* = 96, 67.1%), omental wrapping (*n* = 36, 25.2%), and migration plus omental wrapping (*n* = 11, 7.7%) (Figure 4). The clinical characteristics of the various catheter malfunctions are presented in Table 4. Omental wrapping occurred at a median of 16 days after the operation, while catheter migration occurred later and at varied times, with the longest occurrence time at 7–8 years after the operation. Younger people were more likely to have omental wrapping (mean age, 33.9 ± 13.5 years) than catheter migration (mean age, 40.9 ± 14.7 years) (*P* = 0.014, Table 4).

**Table 4:** Clinical features of PD catheter malfunction.

**Variables**	**Migration (*n* = 96)**	**Omental wrapping (*n* = 36)**	**Migration plus omental wrapping (*n* = 11)**	***P*-value**
Sex (male, %)	57, 59.4%	21, 58.3%	10, 90.9%	0.088
Age (years)	40.9 ± 14.7^*^	33.9 ± 13.5^*^	33.0 ± 12.6	*0.014
Median time of catheter malfunction occurrence (days) (Min, Max)	34.0 (1, 2,840)	16.0 (1, 461)	16.0 (1, 61)	<0.001

* *represented the comparison between the catheter migration group and the omental wrapping group*.

### Analysis of PD Catheter Survival Time

**Figure 5:**
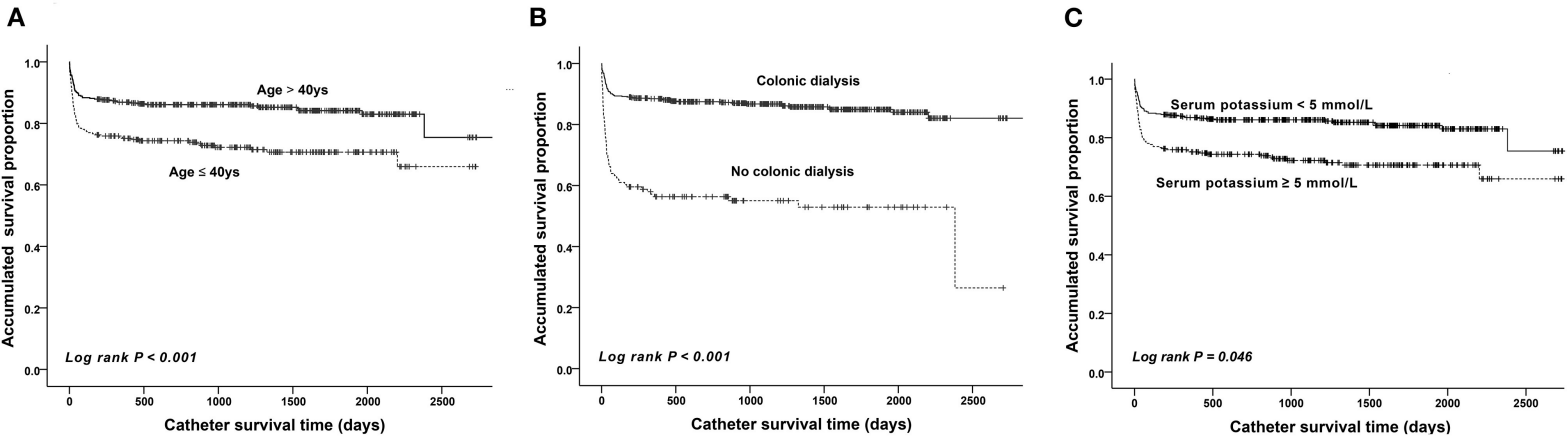
Kaplan–Meier survival curves showing the accumulated age-adjusted catheter survival time **(A)**, colonic dialysis **(B)**, and serum potassium level **(C)**.

The mean follow-up time was 1449.87 days (range, 182–4,374 days). The catheter survival time was available for all included patients. The catheter survival time of the malfunction group (mean, 202.5 ± 479.4 days) was significantly shorter than that of the control group (mean, 1295.3 ± 637.0 days) (*P* < 0.001). Further subgroup analyses were conducted based on age, history of pre-operative colonic dialysis, and pre-operative serum potassium level. The mean PD catheter survival time in patients aged >40 years was estimated at 2382.5 days (95% CI, 2266.9–2498.1 days), which was significantly longer than that in patients aged ≤ 40 years (1955.7 days; 95% CI, 1808.1–2103.3) (*P* < 0.001, Figure 5A). The estimated mean catheter survival time was significantly different between the colonic dialysis (2431.8 days; 95% CI, 2347.2–2516.4) and no colonic dialysis (1408.8 days; 95% CI, 1165.7–1651.9) subgroups (*P* < 0.001, Figure 5B). Moreover, the estimated mean catheter survival time was lower in those with pre-operative serum potassium ≥5 mmol/L (2,382 days, 95% CI; 1761.6–2190.9) than those with pre-operative serum potassium <5 mmol/L (2,840 days; 95% CI, 2205.0–2400.9) (*P* = 0.046, Figure 5C).

### Surgical Complications

After PD catheterization, two patients experienced bleeding. After resting in the supine position, the dialysis fluid was normalized 2–3 days after the operation, with no change in hemoglobin levels. One patient developed peritoneal dialysis-related peritonitis on the first post-operative day. Six cases of exudation at the outlet recovered after 1 week. There were no serious complications, such as visceral injuries or perforations. All patients had good post-incision prognoses, with no occurrence of fat liquefaction or poor healing.

### Treatment Outcome of Catheter Malfunction

Conservative treatment (activity, drainage, manual reduction) (96, 67.1%), second operation (open operation) (42, 29.4%), and extubation withdrawal (5, 3.5%) were used to manage catheter malfunction.

Among the 96 cases of catheter migration, 92 (95.8%) received conservative treatment, three (3.1%) underwent extubation by repeated displacement, and one (1.0%) received a second operation. Among the 36 cases of omental wrapping, one (2.8%) received urokinase sealing, one (2.8%) received extubation withdrawal, and 34 (94.4%) underwent a second operation. Among the 11 cases of catheter migration plus omental wrapping, three (27.3%) received conservative treatment (including urokinase sealing and manual reset), one (9.1%) underwent extubation, and seven (63.6%) received a second operation, of which two underwent three operations. All patients returned to normal after the intervention.

## Discussion

Catheter complications often lead to catheter loss and technical failure. Catheter malfunction has always been a huge burden on PD patients and their caregivers. We found that age, body mass index, pre-operative colonic dialysis, surgeon, and serum potassium level were associated with catheter malfunction in patients with urgent-start PD. Moreover, the catheter survival time of the malfunction group was significantly shorter than that of the control group. Younger age ( ≤ 40 years) and higher serum potassium levels (≥5 mmol/L) may contribute to catheter malfunction, and pre-operative colonic dialysis could reduce the risk of catheter malfunction.

It is often argued that no single implantation approach is the most superior. Regardless of operator performance, in comparing catheter placement by percutaneous needle-guidewire regardless of guided imaging, open surgical dissection, peritoneoscopy, and laparoscopy in identical populations, the outcomes reported were not different (23–28). Local anesthesia for open surgery is considered safe for PD patients, especially for those who are only suitable for local anesthesia/sedation (11). In our center, this is the first choice of insertion for almost all patients. Our clinical experience has proven that this surgical method is safe, reliable, low-cost, and suitable for all patients.

Endogenous personal factors, such as morbid obesity, history of abdominal surgeries, and other diseases such as intestinal diseases, as well as exogenous factors, such as peritonitis and surgical placement technique, have been associated with peritoneal catheter malfunction (29, 30). In our study, younger age, body mass index, and lower surgeon count were independent risk factors for catheter malfunction in patients with urgent-start PD. It has been reported that younger patients were more likely to develop catheter complications in urgent-start PD (7). This is consistent with our finding that patients aged ≤ 40 years were more likely to have catheter malfunction. Young people with rich omenta and active intestinal tracts may be more prone to catheter dysfunction, particularly omental wrapping. For patients with open PD, part of the greater omentum should be resected on a case-to-case basis. The observed redundant omentum lying in proximity to the catheter tip can be displaced from the pelvis into the upper abdomen and either fixed to the abdominal wall or falciform ligament or folded upon itself (omentopexy) (31). However, we did not find the different rates of peritoneal dialysis related peritonitis between younger and older age patients, as well as between colonic dialysis group and no colonic dialysis group, suggesting that peritoneal dialysis related peritonitis was not a distinguishing factor for catheter dysfunction in patients of different ages and in patients with or without colonic dialysis although peritonitis has been reported to be more common in young patients (19). In addition, the technique and experience of the surgeon may contribute to the catheter survival time and success rate (32, 33). Therefore, standardized training for surgeons should be carried out to improve the success rate of catheterization and reduce the incidence of catheter malfunction, thus prolonging both catheter and patient survival time.

Furthermore, pre-operative colonic dialysis was independently associated with reduced catheter-dysfunction risk. A total of 564 (80.6%) patients underwent colonic dialysis before PD catheterization in our hospital. As a semi-permeable membrane, the colon plays an important role in toxic waste removal. The first use of bowel elimination as a treatment of kidney disease could date back to 40 B.C. in Dioscorides' Materia Medica. Later uremic patients were treated with intestinal dialysis or induced diarrhea. Therefore, the colon may provide a therapeutic target for managing CKD (34). Colonic dialysis was reported as one of conservation management for chronic kidney disease patients in stages 3–5 (34–37), but it is not a standard procedure and do not recommend the first-line therapy by guideline. However, whether colonic dialysis is effective in preventing catheter malfunction has not been reported. Therefore, in our center, for patients who choose peritoneal dialysis, we usually only perform colonic dialysis once before peritoneal dialysis catheter implantation according to the patient's will. Subgroup analysis revealed a lower incidence of catheter malfunction and longer catheter survival time in the colonic dialysis group than in the no colonic dialysis group, which are in line with previous findings that good pre-operative bowel preparation is a key step in the success of PD (38, 39). Colonic dialysis can deeply clean the intestines and expel constipation and flatulence, reducing the risk of catheter displacement.

Analysis of blood biochemical indicators revealed only the serum potassium level as an independent risk factor for catheter malfunction. Abnormal blood potassium levels reduce intestinal function, resulting in metabolic waste that cannot be discharged through the anus. This is consistent with the finding that intestinal cleaning before PD catheter implantation may help prevent catheter malfunction. Our results indicated that patients with higher serum potassium levels (≥5 mmol/L) might be prone to catheter malfunction, suggesting that measuring pre-catheterization serum potassium levels may reduce the risk of catheter malfunction.

Of the 107 patients with catheter migration, 96 (89.7%) experienced catheter migration alone, and 92 of them (96%) returned to normal after conservative treatment, indicating its effectiveness in managing catheter migration alone. However, the incidence of omental wrapping accounted for only 5.1% of all patients undergoing catheterization. Although omental folding at the initial open catheter placement can decrease the risk of catheter tip migration with dysfunction (22), we believed that it is not necessary to perform omental folding before open catheter placement under a surgical incision only a few centimeters long, partly because of its difficulty and low incidence. In addition, laparoscopic catheter placement was not superior to open surgery, as the latter required a shorter operative time and simpler equipment (40). In this study, preservation of the peritoneal dialysis tube and the subcutaneous tunnel was successful, suggesting that this operation may be economical and effective in managing omental wrapping.

Our study has several limitations. First, this study is retrospective in nature. Therefore, not all of the desired laboratory data were available for every patient. For the present study, we captured data on a daily basis throughout the hospital stay. These characteristics can reduce associated biases with missing data. Second, some important baseline covariates were not comparable in the original cohort. To reduce the influence of incomparable baseline characteristics, we adjusted the efficacy of PD catheter survival time using multivariate analyses. Third, this was a single-center study. More prospective controlled multicenter studies are needed to validate our findings.

In conclusion, urgent-start PD is a safe and efficacious therapy for patients with unplanned PD. For young people who are prone to catheter malfunction, adequate pre-operative colonic dialysis and serum potassium level control are conducive to preventing catheter malfunction. Moreover, standardized training for surgeons is necessary to reduce the incidence of catheter malfunction. Conservative treatment is effective in managing catheter migration alone, and preservation of the PD tube and subcutaneous tunnel as a second operation is effective for omental wrapping. To our knowledge, this is the largest study on the risk factors and management of PD-related catheter malfunction. This study provides a clinical basis for the prevention and treatment of PD catheter malfunction.

## Data Availability Statement

The original contributions presented in the study are included in the article/supplementary material, further inquiries can be directed to the corresponding author/s.

## Ethics Statement

The studies involving human participants were reviewed and approved by the Ethics Committee of Xijing Hospital. Written informed consent to participate in this study was provided by the participants' legal guardian/next of kin.

## Author Contributions

GX conceived and designed the research, wrote the first draft, and edited and revised the manuscript. LZ participated in data analysis and interpretation. JY participated in all data collection. MB participated in statistical analysis. FD participated in data collection. SS participated in the research design. All authors have read and approved the final version of the manuscript.

## Funding

This study was supported by the National Natural Science Foundation of China (Nos. 81470993 and 81272621) and the Baxter IIS Foundation Grant (Grant No. CHN-RENALIIS-2012-039).

## Conflict of Interest

The authors declare that the research was conducted in the absence of any commercial or financial relationships that could be construed as a potential conflict of interest.

## Publisher's Note

All claims expressed in this article are solely those of the authors and do not necessarily represent those of their affiliated organizations, or those of the publisher, the editors and the reviewers. Any product that may be evaluated in this article, or claim that may be made by its manufacturer, is not guaranteed or endorsed by the publisher.
